# Collateral Victims of Defensive Medical Practice

**DOI:** 10.3390/healthcare11071007

**Published:** 2023-04-01

**Authors:** Ana Cernega, Marina Meleșcanu Imre, Alexandra Ripszky Totan, Andreea Letiția Arsene, Bogdan Dimitriu, Delia Radoi, Marina-Ionela Ilie, Silviu-Mirel Pițuru

**Affiliations:** 1 Department of Organization, Professional Legislation and Management of the Dental Office, Faculty of Dental Medicine, “Carol Davila” University of Medicine and Pharmacy, 17-23 Plevnei Street, 020021 Bucharest, Romania; 2 Department of Prosthodontics, Faculty of Dental Medicine, “Carol Davila” University of Medicine and Pharmacy, 17-23 Calea Plevnei, 010221 Bucharest, Romania; 3 Department of Biochemistry, Faculty of Dental Medicine, “Carol Davila” University of Medicine and Pharmacy, 17-23 Plevnei Street, 020021 Bucharest, Romania; 4 Department of General and Pharmaceutical Microbiology, Faculty of Pharmacy, “Carol Davila” University of Medicine and Pharmacy, 6 Traian Vuia Street, 020956 Bucharest, Romania; 5 Department of Endodontics, Faculty of Dental Medicine, “Carol Davila” University of Medicine and Pharmacy, 17-23 Plevnei Street, 020021 Bucharest, Romania

**Keywords:** defensive medicine, malpractice, professional error

## Abstract

This paper analyzes the phenomenon of defensive medical practice, starting from the doctor–patient relationship, and the behavioral and professional factors that can influence the proper functioning of this relationship and the healthcare system. We analyze medical malpractice, given the increase in the number of accusations, as an essential factor in triggering the defensive behavior of doctors, together with other complementary factors that emphasize the need for protection and safety of doctors. The possible consequences for the doctor–patient relationship that defensive practice can generate are presented and identified by analyzing the determining role of the type of health system (fault and no-fault). At the same time, we investigate the context in which overspecialization of medical personnel can generate a form of defensive practice as a result of the limiting effect on the performance of a certain category of operations and procedures. The increase in the number of malpractice accusations impacts the medical community—“the stress syndrome induced by medical malpractice”—turning doctors into collateral victims who, under the pressure of diminishing their reputational safety, practice defensively to protect themselves from future accusations. This type of defensive behavior puts pressure on the entire healthcare system by continuously increasing costs and unresolved cases, which impact patients by limiting access to medical services in the public and private sectors.

## 1. Introduction

Healthcare systems consist of a series of elements that characterize the general management framework of all existing relationships. There are two main actors in a healthcare system—the doctor and the patient—who are affected by the rights and obligations that regulate their relationship. Doctors, based on the skills and abilities acquired by completing medical studies, obtain the title of doctor/specialist and the right and ability to provide and communicate information within the limits of the acquired skills [1]. The quantity, quality, and veracity of such information are directly proportional to a doctor’s level of awareness of the need for continuous development and learning [2,3,4], a process that must guide the professional path of each doctor.

Despite the complex training that must be completed, medical activity, like any other type of activity, is susceptible to professional errors [5] that affect the professional futures of medical staff in the light of their different experiences [6].

This paper focuses on the consequences of a potential malpractice accusation, whether it has led to a court case or, in the absence of litigation, it is based on the constitutive conditions of medical malpractice that have not been met. We analyze a phenomenon that has recently reached a large scale, impacting the entire medical community because of the risk of triggering a mechanism that is likely to disrupt the healthcare system—defensive medicine [1,7,8,9]. The results of defensive medicine place doctors in the position of being collateral victims of this phenomenon [10], and with a boomerang effect—opposite to what might be expected—limits patients’ access to medical services [11,12].

## 2. Materials and Methods

This article is based on research over a period of nine months, from May 2022 to January 2023. The research aimed to identify and analyze studies and reports on medical malpractice, the continuously increasing number of malpractice cases, the legal regulation of fault and no-fault systems, and the phenomenon of defensive medicine. The included reports were completed by the following institutions/organizations: the American Medical Association; the National Practitioner Database, Department of Health and Human Services (USA); the Physician Insurers Association of America; the Department of Health and Social Care (UK); the National Agency for Regional Health Services (Italy); the Civil Court of Rome, Italy; and the National Office for Therapeutic Accident Compensation (France). 

The data presented in these reports were used to generate a comprehensive picture of all the factors that trigger defensive medicine and the consequences of this phenomenon for doctors, patients, and the healthcare system. We also provide viable solutions for reducing this phenomenon. 

### 2.1. Malpractice in the Healthcare System Context

Professional medical activity is special due to the spectacular results it can generate. Doctors are sometimes associated with ancient deities, creating a symbiosis between the rational and the emotional, affecting the rigidity and correctness of applying medical knowledge and the ability to effectively relate to patients. The harmony of the doctor–patient relationship is susceptible to numerous influences and factors that can disrupt its correct course, generating a cascade of damage for the involved participants.

Starting from this relationship and its supreme social value for “human life and health” [13], international regulations and Romanian laws established specific instruments to govern the professional behavior of doctors, consistent with the legislative benefits that are guaranteed for patients [13,14,15].

Under the rigous of the law, doctors are responsible for professional errors made during medical activities. Professional errors that cause damage to a person subject doctors to legal liability. Given the nature of the medical profession, Romanian law has been established in terms of protecting doctors and injured patients, starting from the premise that the intention and the purpose of a doctor’s action is to support and help a patient in solving a particular medical problem.

**Figure 1:**
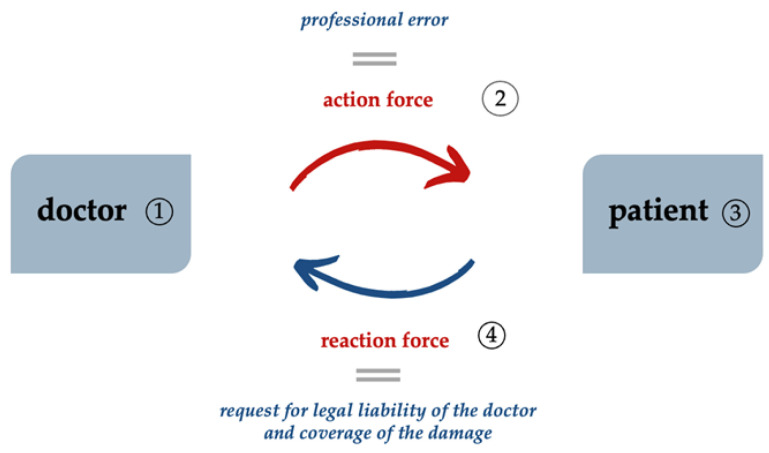
The third principle of mechanics—the “Principle of Action and Reaction”—applied to the doctor–patient relationship.

Professional errors committed in the exercise of medical activities within the doctor-patient relationship can be considered by applying the third principle of mechanics—the “Principle of Action and Reaction”—i.e., when one body acts on another body with a force (called the action force), the second body also acts on the first body with a force (called the reaction force), having the same magnitude but an opposite direction. In this sense, we note the existence of two “bodies”—i.e., the doctor and the patient. The medical action (1) in which a doctor makes a professional error (force of action) (2) generates the patient’s reaction force (3); the patient identifies the damage and the reaction force is equal to the patient’s decision to fight back and seek legal liability in a court and/or compensation for the damage (4) (Figure 1).

If the conditions required by law are met (*the illicit act, the damage, the causal link, and the guilt*), a court will find the existence of an act of malpractice and compensate an injured patient [16]. 

However, the law introduces a new actor—the insurer—to assist both the wrongdoing physician or surgeon and the injured patient in paying for the claimed compensation, through professional malpractice civil liability insurance. In situations of medical malpractice, the insurer will cover the damage caused to the patient by medical error and pay the damages determined by a court decision.

An exception to this situation is the occurrence of an “ethical” error, characterized by non-compliance with applicable legislation on medical ethics (e.g., lack of informed consent, non-respect of confidentiality, exceeding the limits of competence, limiting access to medical services, or limiting access to a medical file) [14]. In such cases, the insurance contract’s protection mechanism will not work and the payment of damages will be made by the doctor.

As we have noted, the doctor–patient relationship is not a relationship that is independent of rules and norms; rather, it is integrated into a complex health system. In terms of medical malpractice, a healthcare system is characterized by a series of fundamental principles that underlie the legal system that governs all the existing relationships in a society. As legislation defines and shapes various aspects of professional activity, it is important to identify the elements that underlie the analysis and evolution of malpractice in different parts of the world while, at the same time, outlining the effects and consequences that directly affect the doctor–patient relationship and the entire health system.

In considering medical malpractice, two types of health systems can be identified, which differ in terms of characteristics and functionality—**the fault system** and **the no-fault system** [17,18] (Table 1).

The adoption of one type of system instead of the other reflects the financial strategy and relational management applied at the state level with respect to the attitude and management of medical malpractice conflicts [17,18]. These considerations can be analyzed from the perspective of studies on the number of malpractice cases, their evolution over time, and associated costs. The phenomenon of medical malpractice greatly reconfigures a community’s perception of error in the healthcare area when there is a continuing increase in the reported number of patients who were possibly harmed by a medical act. In the UK, the Department of Health and Social Care reported significant increases in the number of malpractice allegations: in 2006/2007, there were 5426 claims, compared to 2021/2022, when there were 15,078 claims [19]—a percentage increase of 177%. In Japan, malpractice cases brought to court increased from 400 in the 1980s to 1110 in 2004 [20]. In the US, the number of medical malpractice lawsuits increased by 300% between 1965 and 1970 [21]. In the last 20 years, the number of malpractice litigation cases has decreased slightly, according to the American Medical Association (AMA)—there were 2146 cases in 2006, compared to 1903 cases that were registered and settled in 2015 [22]; however, malpractice allegations were driven by large payouts to injured patients—the average claim payout per case was $365,503 in 2015, a 10.7% increase compared to 2006 [22]. 

It is important to note that the litigation numbers presented by the AMA correspond to 7% of the total claims made by patients in 2015—i.e., cases resolved in court [22]. The National Practitioner Database, managed by the US Department of Health and Human Services, produces annual reports on the status of malpractice payments, including court-ordered payments and out-of-court settlement payments. The 2019 report made, which presented a comparative analysis of the payments made between 1991 and 2017, showed a decrease in the number of recorded payments and a constant increase in the average payment [23]

An analysis carried out in Rome, Italy, during the five years between January 2016–December 2020 showed that the Civil Court of Rome considered 1117 malpractice cases [24]. From January 2018 to February 2019, 280 cases regarding legal liability for malpractice were submitted to trial, representing about 20% of all national disputes [25]. The total amount paid was EUR 23,489,254.08, with an average payment of EUR 163,119.82 [25].

In 2008, Harvard studies estimated that the annual costs associated with medical malpractice in the US were $55.6 billion, which was equivalent to 2.4% of the total national healthcare expenditures [26].

An increase in the number of medical malpractice cases (200–500%) was also recorded in the Baltic States, the Eastern European States (50%), Germany, Italy, the countries of the Iberian Peninsula, and the countries in the Mediterranean Sea area [27].

These statistics refer to states that have adopted the fault system, which is focused on promoting conflict by postponing compensation for injured patients until a doctor’s guilt is proven. This system generates repercussions for the entire health system, as well as for doctors and patients.

In comparison, the no-fault system, which provides for the active mediation of conflicts by promoting settlement via extrajudicial procedures to satisfy injured patients, is characterized by the very small number of cases that are settled before the courts, as shown by relevant statistics: 0.1% in Sweden, 0.3% in Finland, and 0.5% in Denmark [28].

In Sweden, which adopted the no-fault system in 1975, more than 90% of claims for compensation by injured patients are processed via extrajudicial methods, through the Patient Insurance Association. Of these cases, only 45% of the processed requests achieve a positive result in terms of compensation [29].

Since 2002, France has been characterized by the implementation of a mixed system (fault and no-fault). The French system is based on the principle of solidarity, whereby injured patients have the right to address the Regional Conciliation and Compensation Commission, which, through the National Office for Therapeutic Accident Compensation (ONIAM), determines the coverage for the damage created [30,31]. If a patient is not satisfied with ONIAM’s determination, the patient has the right to go to court. ONIAM’s 2021 activity report showed that 96% of the decisions were accepted by injured patients, which meant that only 4% of patients asked courts to resolve the conflicts. In addition, in 2021, the average value of compensation paid per case was EUR 142,500 [32], which was almost half the average compensation per case paid in the USA.

The exponential increase in the number of lawsuits regarding the legal liability of doctors for making a possible error in the application of a treatment, as well as the high costs incurred to cover the alleged damages, are the foundation of a developing phenomenon in the medical community—***defensive medical practice***. 

The growing trend of patients who were possibly harmed by an act of malpractice to pursue burdensome complaints against their doctors, a specific characteristic of states that have adopted the system based on proof of error (fault system)—creates an oppressive pressure on the professional life of doctors, generating fear and tension in doctors’ relationships with patients because of the possibility of a malpractice charge.

**Figure 2:**
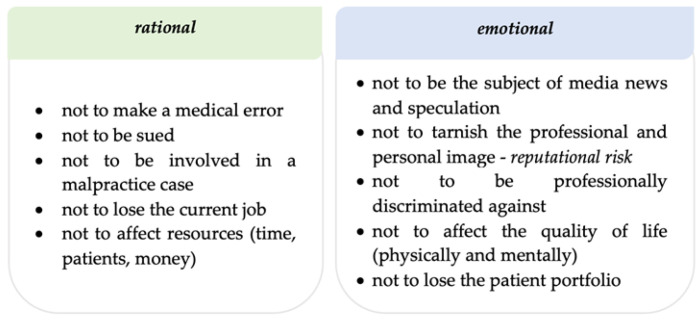
Elements that generate negative impacts from doctors’ emotional and rational perspectives.

This increase in medical malpractice accusations is the main triggering factor for the defensive practice phenomenon, in terms of the elements that generate negative impacts from doctors’ emotional and rational perspectives (Figure 2).

### 2.2. Defensive Medicine—History

To analyze the defensive medicine phenomenon, we start from a series of definitions from the specialized literature. Defensive medical practice (DMP) is a set of specific behaviors pertaining to clinical actions in order to protect physicians or surgeons against litigation or adverse outcomes [1,7,8].

DMP may also be characterized as a series of medical actions taken by doctors, in disregard of the mandatory expectations provided by applicable recommendations and specialist guidelines, which are intended to function as a shield to protect doctors against professional negligence complaints and possible accusations of malpractice by patients or their relatives [7,8,33].

Historically, the phenomenon of “defensive medicine” was first identified in the early 1970s in the United States of America. The concept gradually expanded to Europe, according to a 2020 review of articles analyzing the phenomenon [33]. The first mentions of DMP and its scope are found in public speeches of the General Counsel of the American Medical Association. In 1974, the General Council established a first approach to DMP in a positive sense, urging medical practitioners to reduce the risks to which they may be subjected due to possible accusations of malpractice. The recommendations covered two types of positions that could be adopted by doctors:

1. *Do not perform any surgery, do not prescribe or administer medicines, do not touch the patient, do not perform any manipulation, and pray a lot, or*
2. *Practice medicine defensively [34].*

At that time, the DMP phenomenon was presented more as an intrinsic need of the medical profession, an attitude that was justified in terms of the high risks to which medical workers were subjected, without emphasizing the implied repercussions. At the same time, there was a big difference between recommending additional tests for the benefit of patients and carrying out tests that were unnecessary or useless at any stage during the doctor–patient relationship [34].

Over time, studies that analyzed the functionalities and elements that trigger defensive medicine at the medical community level have confirmed the definition of DMP provided by the specialized literature: i.e., a doctor carrying out medical activities in excess or not doing what the doctor should do, in order to reduce the risk of a possible accusation of malpractice, thereby generating negative effects on the doctor’s professional and personal life, physical health, or mental health.

In general, the specialized literature distinguishes two kinds of DMP: “positive” and “negative”.

**“Positive” DMP** is characterized by a physician’s or a surgeon’s action in prescribing and recommending unnecessary and repetitive additional tests/procedures [33,35], recommending tests in situations where the risk of disease is very low and the test does not contribute to the correct diagnosis, or sending a patient to another specialist doctor for a consultation or complicating the treatment without justification and need, in order to avoid an accusation of malpractice [1].

**“Negative” DMP** is the physician’s or surgeon’s attitude in not dealing with high-risk patients or performing risky procedures, as an avoidance position [11,36].

### 2.3. DMP Statistics

**Figure 3:**
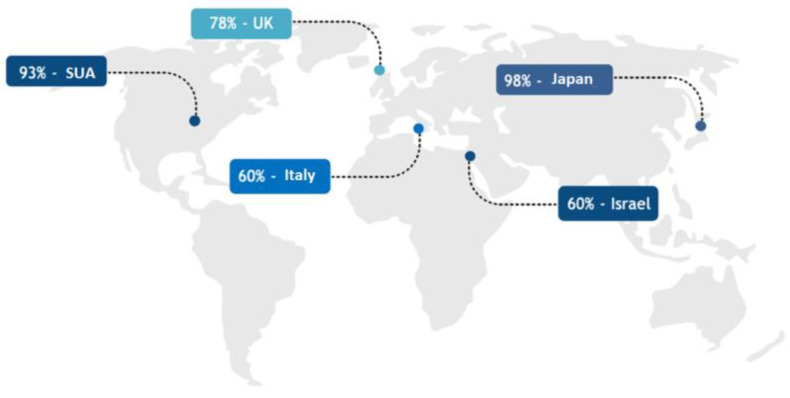
Statistical data on DMP.

Studies conducted in different countries show a worrying increase in the phenomenon of DMP (Figure 3). For example, according to a study conducted in 2003 in the American state of Pennsylvania, 93% of the 824 participating doctors admitted that they practice defensively. The results of that study highlighted certain DMP-specific actions performed by physicians or surgeons: use of imaging technology in unnecessary circumstances (43%) and restriction of medical activity in terms of eliminating procedures that were susceptible to complications (42%). Moreover, doctors attributed their defensive behavior to a lack of confidence in the effectiveness of professional liability insurance [36].

Another study that confirmed these results was carried out in America in 2012, at the national level, in which 96% of the respondents (orthopedic doctors) admitted that they practiced defensive medicine in a positive way [37].

The situation is similar overseas. A study carried out in Great Britain in 2013 indicated that 78% of doctors acted defensively, and the most “popular” form of DMP was the recommendation of unnecessary tests [7].

In Israel, a four-month study in 2008 highlighted a potential trigger for doctors’ defensiveness: 40% of 889 doctors approached patients as a threat and 60% of the doctors reported defensive actions [38].

In 2014, the National Agency for Regional Health Services in Italy published the results of a study that identified both the degree of practicing defensive medicine (58% of respondents practiced DMP) and doctors’ personal assessments regarding the evolution of this phenomenon (93% of respondents considered that DMP would increase and 64% were certain that the safety offered by DMP, would cover the risk of a medical error) [39].

In 2006, a Japanese study reported worrying results:98% of the participating doctors declared their active practice of defensive medicine, with a predominant emphasis on an avoidance attitude and reconfirmation and reassurance regarding a given diagnosis [40].

## 3. Results and Discussions

In the previous sections, we considered the elements that impact the doctor–patient relationship, as well as the complexity and uncertainty such elements present, such as the increasing number of malpractice cases, the increasing payment amounts in malpractice cases, the types of healthcare systems in terms of legal regulation, and the development of the phenomenon of defensive medical practice.

Considering all these elements, our personal approach to the phenomenon of defensive medicine has been designed, outlining and generating a comprehensive picture of all the factors that underlie the triggering of defensive medicine, the consequences of this phenomenon, and viable solutions for reducing this phenomenon.

### 3.1. A Cascade of Factors Justifying DMP

As we discussed earlier, the main factor that triggers the defensive attitude of doctors is the continuing increase in the number of malpractice complaints, as well as the payments of increasingly large amounts for material and emotional damage to patients. This tendency can generate a deep sense of fear [8] that causes the **clinical–judicial syndrome** (CJS) for physicians and surgeons.

*The clinical–judicial syndrome* was defined for the first time in 1993 by Dr. Elias Hurtado-Hoyo (Medical Association of Argentina), who stated that a doctor’s activity is considerably affected when the doctor is involved in litigation [41].

CJS can be extended to other areas that make doctors vulnerable, including extrajudicial areas. Considering the personal characteristics of each doctor, this syndrome can arise upon the initiation of a judicial/extrajudicial procedure, in the stage of settling the case (via mediation, the commencement of court proceedings, the hearing from parties and witnesses, the communication of the decision, appeals from the decision, etc.), and/or after the completion of the procedure (e.g., the execution of a court decision). The clinical–judicial syndrome involves a series of behavioural, physical, and mental changes in a doctor, including asthma, headaches, diarrhea, immunodepression, anxiety, paranoia, sexual dysfunction, isolation, drug and alcohol use, and insomnia [10].

Litigation regarding doctors’ liability results in professional risk, and the specialized literature has identified another concern applicable to this area—the stress syndrome induced by medical malpractice (SSMM) [42].

We consider that these syndromes (CJS and SSMM) not only target doctors who have suffered as a result of litigation, but also doctors whose colleagues have been involved in a legal process, upon witnessing their suffering and that of their family members.

At the same time, the defensive attitude could be justified based on **the psychological perspective of an individual’s need for protection**. The fear of a potential legal process as a result of harming a patient induces a state of constant caution in a doctor, making the process of treating the patient and the doctor–patient relationship much more difficult. Thus, a doctor may resort to an “effective” method of protection—defensive medicine. Such actions taken by a doctor can be analyzed by means of the psychological concept of “fight or flight”—the behavioral reaction of an individual in response to stress—concept defined by Professor Cannon in 1915 [43]. Over time, the concept has been expanded to a broader “fight, flight, freeze or fawn” model, by which we understand a person’s fight, flight, freeze, or fawn response [44].

**Figure 4:**
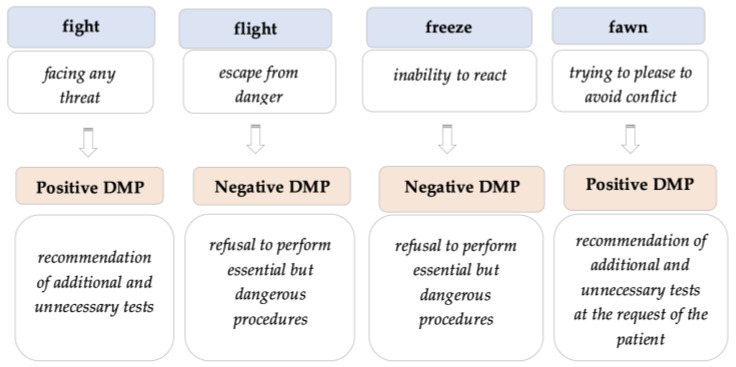
Concept of the “four Fs” related to a doctor’s reaction to stress.

If we consider the “four Fs” in relation to a doctor’s reaction to stress as a foundation of defensive practice, we can identify the following connections *(*Figure 4*)*:

The main reason for DMP is the fear of being sued by a patient who has noticed damage that occurred as a result of an applied treatment, accompanied by the fear of a large payment to cover the emotional and material damages. It is a very unpleasant situation, generating anxiety, uncertainty, and insecurity. However, it is important to bear in mind that we are talking about doctors who are specialists and professionals in their fields.

The very existence of a claim by a patient suffering from possible harm is alarming, without the additional anxiety of legal proceedings.

However, it is interesting to analyze how justified a doctor’s defensive attitude may be in the context of the number of cases that are similar to the one involving the doctor. There are few documents that analyze this aspect. 

We refer to two reports based on the information collected through two studies carried out by the American Medical Association (AMA), involving data provided by the Physician Insurers Association of America (PIAA). These studies, in 2008 and 2015, generated important information regarding the level of legal liability of doctors in the US, analyzed the number of claims made by allegedly injured patients, how many of these claims developed into lawsuits, how many of these claims were resolved through alternative, extrajudicial methods, and how many of these claims were rejected on the ground of not meeting the constitutive and evidential elements of damages that would lead to the physician’s or surgeon’s liability. These two studies allow for a comparative analysis, in light of the seven-year difference in which they were completed.

**Figure 5:**
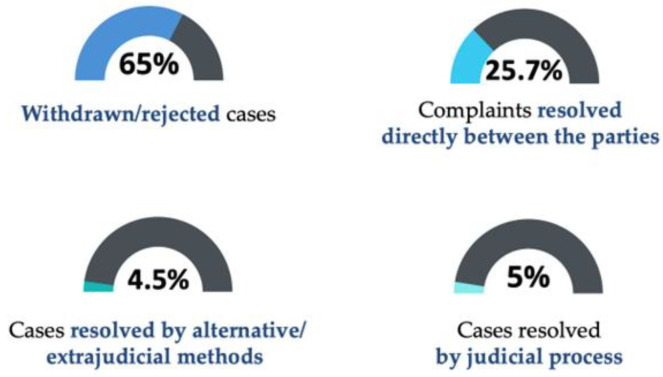
Statistical data regarding malpractice liability, USA, 2008.

In 2008, the PIAA presented the following statistical data regarding of professional malpractice liability insurance: of the total number of cases analyzed, 65% were rejected as unfounded or were withdrawn by the patients; 25.7% of the applications resulted in a solution directly between the doctor and the patient; 4.5% of the cases were resolved by extrajudicial methods; and *5% of the claims followed a procedure that was specific to the courts* [45] *(*Figure 5*).*

**Figure 6:**
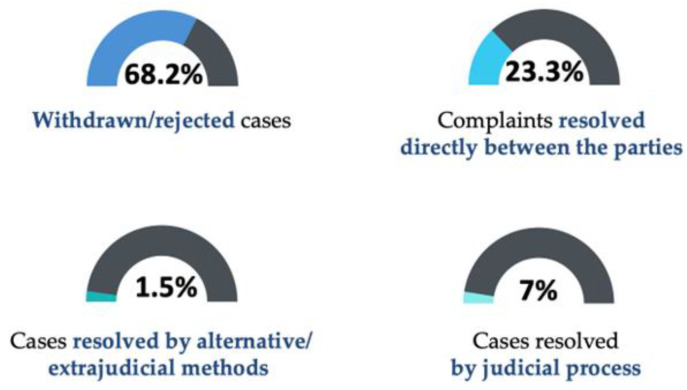
Statistical data regarding malpractice liability, USA, 2015.

In 2015, another study based on the same evaluation indicators showed the following: 68.2% of the claims made were rejected/withdrawn; 23.3% of the claims were resolved directly; and *7% of the claims were resolved before a court (*Figure 6*)* [22].

**Figure 7:**
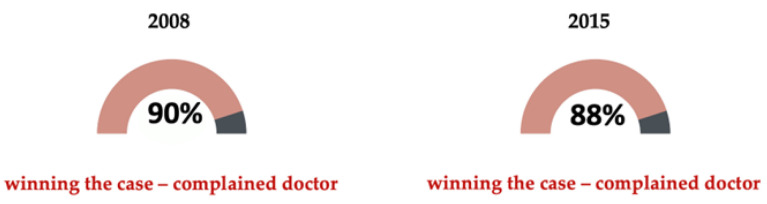
The percentage of cases won by doctors in 2008 and 2015.

Of the total number of claims that reached a court— 5% in 2008 and 7% in 2015—the vast majority resulted in decisions in favor of the doctor (90% in 2008 and 87.5% in 2015) [45] (Figure 7*).*

In this article, we are less interested in the number of claims made by allegedly injured patients; we are more interested in analyzing the route taken to resolve a claim and the finality of claims, focusing on the two main actors involved.

The **patient**’s right to justice is acknowledged, protecting important constitutional, social, and personal values—i.e., the protection of the health, physical integrity, and mental integrity of the individual. The patient’s right to justice includes the patient’s ability to use any legal means to request compensation in cases of possible harm. The right to justice involves access to institutions that are empowered to resolve acase by determining the identity of the wrongdoer, the prejudicial act, the guilt, and the connection among these factors. The statistical data set out above confirm the functionality of the judicial system, in terms of respecting the right to file a complaint and/or to withdraw a claim.

On the other hand, from the **doctor**’s point of view, the previously mentioned legal norms must be consistent with the provisions of the Universal Declaration of Human Rights, which in Article 7 and Article 10 require equality before the law, equity, independence, and impartiality of courts [13].

Based on the data provided by the reports above and by aspects of DMP that stem from physicians’ and surgeons’ fears of being embroiled in lawsuits about alleged harm to patients, we can conclude that physicians and surgeons overestimate the risk of a malpractice charge that would result in their legal liability, considering that the PIAA reports in 2008 and 2015 showed that approximately 90% of cases that reach the courts end with decisions favorable to the doctors.

This unjustified defensive attitude, based on the number of cases in which doctors are accused, could be explained by the application and reinterpretation of **the Pareto Principle—the rule of the few but critical** or **the 80/20 rule**—which states that for many events, approximately 80% of the effects are produced by 20% of causes [46]. Applying this principle to the phenomenon of medical malpractice as an essential factor in triggering defensive medical practice, together with the fact that the majority of court cases result in doctors being acquitted, we understand that a minority of cases in which doctors are accused have the negative impact to produce an unjustified maximum of effects, promoting the tendency for doctors to practice defensively.

**Figure 8:**
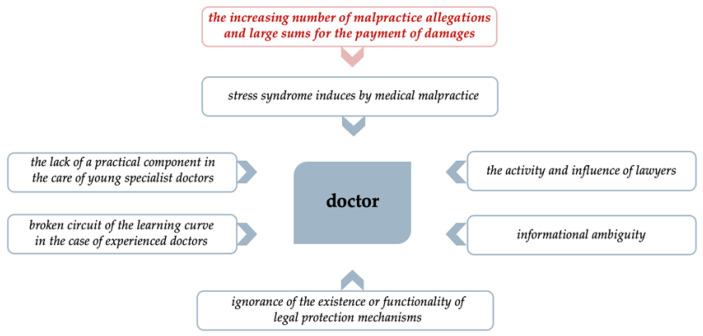
Complementary factors that trigger the defensive attitude of doctors.

Although the statistics are advantageous for doctors, which should mitigate the feeling of fear of malpractice accusations, other ***complementary factors*** intervene (Figure 8) that trigger doctors’ defensive attitude, as set out below:

- ***the lack of a practical component in the case of young specialist doctors***—in accordance with the *“learning curve”* concept, young specialists are the quadrant characterized by *“awareness of the competence”* they have, as a result of the studies they have carried out and the testing and certification of their acquired knowledge. However, their lack of extensive practical experience may increase their need for extra caution, due to fear of possible error;
- ***the broken circuit of the learning curve in the case of experienced doctors***—the era in which we live, learn, and work professionally is defined by continuous technological and informational progress, which “forces professionals”, through the complexity of the world, to be updated to present requirements in anticipation of the rigors expected in the near future. This circuit of the learning curve places highly experienced doctors in the category of *“competency unaware”*, which is characterized by a high level of automatism in the professional activity they carry out. The interruption of the learning curve is caused by reluctance to adapt to the new [4].
- ***ignorance of the existence or functionality of legal protection mechanisms***—among legal protection mechanisms, we include *informed patient consent*, which provides protection for both doctors and patients, ensuring the implementation of doctors’ legal obligation to communicate and provide information and respecting the principle of patient autonomy. Another legal protection mechanism is professional civil liability insurance for medical malpractice, which provides a safety net for doctors by covering damages caused by medical errors, as well as a safety net for patients by ensuring recovery for the damage caused.
- ***informational ambiguity***—the medical profession needs to build and maintain a good reputation. The press, through its existing means of communication, can significantly affect professional reputations. For this reason, doctors practice defensively to avoid negative publicity, staying in a reputational safety zone. The media can also have a very important impact on patients by increasing their tendency to see themselves as victims of medical malpractice and by presenting the most extreme cases in newspapers and on TV [47]. Thus, in the absence of specialized medical knowledge, patients can be easily influenced by articles without scientific and statistical data pertaining to correct medical practice, and be stimulated to blame medical staff unjustifiably.
- ***the activity and influence of lawyers***—legal professionals can significantly affect the normal course of medical activity, first by supporting proceedings before courts to prove the guilt of a doctor, and second by promoting patients’ sense of victimization and their belief in the success of cases in court. In addition, an important element is the growing number of lawyers who expand their practices to areas with high economic potential based on the importance of protected values.

### 3.2. Overspecialization—A Possible Form of DMP

Considering the multitude of factors previously analyzed in this article, doctors are in a situation where they have **a deep need to adapt to the health system and to the existing mechanisms for managing malpractice cases** in order to minimize the risks associated with the profession and to face the social pressure of patient expectations. In certain contexts, this adaptation could cause dentists, specialist dentists, and specialist doctors to overspecialize.

Considering the normative acts that regulate the activities of the dentists, specialist dentists, and specialist doctors, the skills acquired as a result of progressing through the elements of learning cannot be negated dby overspecialization in certain areas of practice. Thus, the capacity of doctors to practice across the entire spectrum of competencies conferred by law is expected, without limitation to areas of over-specialization.

**Figure 9:**
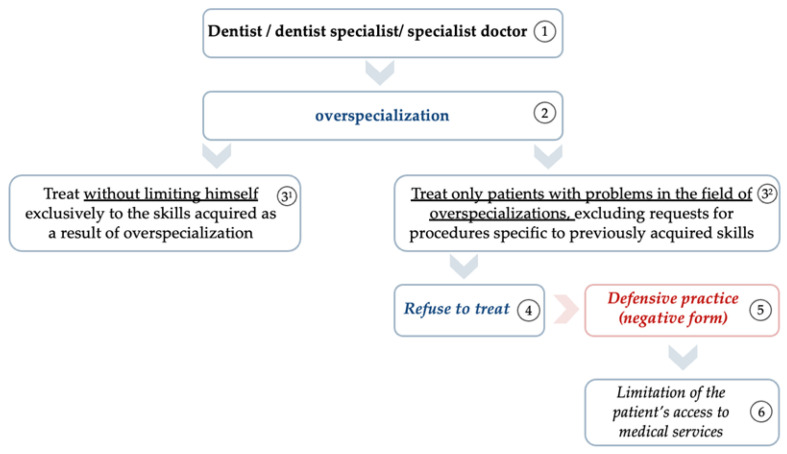
Overspecialization interpreted as a possible negative defensive practice.

In specific situations (excluding those in which dentists, specialist dentists, and specialist doctors decide to follow an overspecialization program to justify their passions for a certain type of practice) this decision can sometimes reflect a conscious reaction to exclude the possibility of a future error and to remove the fear of a possible mistake by acquiring an impressive level of automatism in a certain field. This reaction could be interpreted as a possible action specific to a negative defensive medical practice, as illustrated in Figure 9.

Thus (with reference to Figure 9), dentists, dentist specialists, and specialist doctors (1), as a result of completing an overspecialization program (2), in their relationships with patients, can adopt two types of behavior: *treating patients without limiting themselves exclusively to the skills acquired as a result of overspecialization (3^1^)* or *treating only patients with problems that fall within their field of overspecialization, excluding requests for procedures specific to previously acquired skills (3^2^).* The second type of behaviour can be manifested by the medical practitioner’s action in unjustifiably refusing *(4)* to perform certain medical procedures for certain patients, although such procedures are within the practitioner’s competence. In this way, the medical practitioner directly limits the patient’s access to medical services *(6)* by performing defensive medical practice in a negative form *(5)*.

Once again, such a professional attitude can be considered a defensive medical practice only if the decision to overspecialize and perform medical procedures exclusively in a limited sphere comes from the desire to develop a considerable level of automatism within a certain practice area to minimize the risk of making an error, and to remove accusations of malpractice and decrease the risk of professional damage to the medical practitioner.

### 3.3. The Consequences of DMP

DMP is a phenomenon that involves a series of consequences, which we analyze from the perspective of the *doctor–patient* relationship, focusing on the impact that relationship has on each actor. We also consider the effects DMP has on *the healthcare system*.

From the ***patient***’s perspective, as a beneficiary of healthcare services, we can identify the following consequences:

▪ *Limiting the patient’s access to medical services* by the doctor’s refusal to perform essential but dangerous procedures—practicing negative defensive medicine.
▪ Being dominated by feelings of *distrust and insecurity about medical decisions* due to the doctor’s actions in recommending additional and unnecessary tests (positive defensive practice). Thus, the costs of carrying out treatment increase significantly, thereby increasing the risk of abandoning a proposed treatment and leading to possible degradation of the patient’s health and requiring greater financial resources in the future for the patient’s recovery.

From the perspective of a ***doctor*** who practices defensively due to the need to defend against possible malpractice accusations, we observe the following consequences:

▪ The continuous increase in fear of malpractice allegations generates the doctor’s instability and insecurity, which influences and diminishes the doctor’s *reputational safety*. Doctors can cover payments for damages caused by a medical error by purchasing a professional liability insurance policy, but such a document tht does not have the capacity to ensure the doctor against the psychological costs and the stress caused by involvement in litigation or the reputational effects of a lawsuit (reduced income, damaged status) [26].
▪ *Decreasing the efficiency of the medical actions* performed by the doctor through positive defensive practice, in terms of increasing the complexity of medical interventions.
▪ *Degradation of the doctor’s relationship with the patient* by increasing the time needed to identify and resolve the patient’s needs, requiring additional specialist investigations and consultations.

Starting from the idea that the doctor–patient relationship represents the basis of any ***healthcare system***, the degradation of this relationship through of DMP has immediate consequences on the entire system:

▪ *Increasing pressure on the system* due to its inability to ensure the correct management of the relationship between the doctor and the patient.
▪ *The continued increase in costs of resolving medical cases* as a result of the need to protect the doctor by requesting additional specialist investigations and consultations. The impact that defensive medicine has in terms of increased costs has been analyzed in the specialized literature—US 12 billion 1987 and a doubling of these costs in 1997 [48]. In 2008, a Harvard University study showed estimated costs of US 45.6 billion associated with defensive medicine [26].
▪ *Decreased patient access to medical services* as a result of increased costs. From the perspective of financing health services via funds allocated from national budgets, defensive practices treat a smaller number of patients with the same financial resource. In private healthcare systems, in which the patients contribute from their own income to pay for necessary treatment, fewer and fewer patients will be able to afford certain treatments as a result of the continuous increase in costs. The increase in the costs of resolving medical cases as a result of the unjustified use of health services leads to the blocking of human resources by involving a larger number of medical personnel and the rapid consumption of allocated financial resource. These two elements, which are commonly found due to defensive medical practice, limit the access to medical services by patients who have a great need for medical assistance [12].

### 3.4. Solutions to Diminish the DMP

In order to reduce the phenomenon of DMP, it is necessary to identify effective and applicable solutions at the level of the two main actors involved and at the level of the legal and health systems that encompass and regulate the doctor–patient relationship.

From the **patient’s** perspective, the solutions start from an awareness and an assumption of the double mission and role of doctors, as the main protagonists who have the legal competence to direct and guide the medical course of patients:

▪ the role of the doctor to treat the patient is fulfilled by the doctor’s mission to contribute to the correct education of the patient by promoting a positive attitude regarding prevention and an approach to a healthy lifestyle, ensuring continuous information that enables the patient to be aware of the characteristics and components of appropriate medical care as a complex medical process [49,50].
▪ realizing the connection between education and treatment, the doctor will have the ability to build a correct relationship with the patient, based on trust in the doctor’s professional skills [50].
▪ in order to reduce any conflictual tendency of a patient, there must be certainty regarding damage recovery following an act of malpractice, with an understanding by the patient of the legal mechanisms that cover the damage caused, including professional civil liability insurance.

From the perspective of a ***doctor*** who practices defensively, the solutions include the following:

▪ the need to amplify *the doctor’s role in the process of educating patients*, starting from the youngest ages.
▪ *reducing the reputational risk* by developing and increasing the doctor’s reputational mentality. The increase in reputational safety can be achieved by training the doctor’s *critical thinking*, which represents the fusion of the doctor’s *optimism* in continuing to practice and *the ability to analyze the pitfalls*, in terms of being aware of the certainty and the extent of risks that influence the doctor’s activities (as most malpractice cases absolve the doctors of liability).
▪ transforming the negative approach into a need to be aware of the circumstances in which an error occurred, analyzed from the perspective of the *learning opportunity* [5].
▪ the need for clarity and vision regarding the complexity of professional activity. This can be achieved by ensuring a perpetual circuit of the learning curve [3,4], which would allow the periodic adaptation of the professional in response to new demands of the profession. Doctors need to understand and interpret legislative norms, acquire of soft skills (communication, emotional intelligence, time management, etc.), master digitization, understand cost-efficiency-based management, etc. It is recommended that the defensive, self-protective behavior be replaced with continuous learning [2,3], regardless of the speciality and the accumulated work experience, supplementing such learning with an important component of interpersonal empathy, which would increase the safety of patients [48].

In discussing solutions that would improve the functionality of ***the healthcare system***, we believe that ensuring a favourable environment for the provision of medical services and minimizing the many economic, social, and legislative pressures would facilitate the creation of a safety zone for doctors and reduce the risk of relationship degradation with the patient:

▪ the redistribution of “legislative attention” from the severity of punishment for professional error to the satisfaction of the injured patient, relying on the replacement of the punitive attitude with a need to identify and correct the errors [51], specifically within ***the no-fault system***.
▪ Exchanging legislative and informational uncertainty for clarity and understanding of the system level’s steps taken, in terms of orienting the social mentality toward eliminating the concept of blaming someone who made a mistake [52] and supporting that person in order to report the error immediately.
▪ A possible solution to the treatment of defensive practice is the use of ***artificial intelligence as a second opinion tool***, functioning as an element of social proof based on statistics, thereby diminishing the reputational competition of doctors as medical services providers. Artificial intelligence, from the perspective of providing a second opinion, would provide an ally for doctors in terms of the correctness of diagnoses and proposed treatment options based on information and statistical data, thus reducing the tendency of doctors to practice defensively by adding tests or refusing patients.

## 4. Conclusions

**Figure 10:**
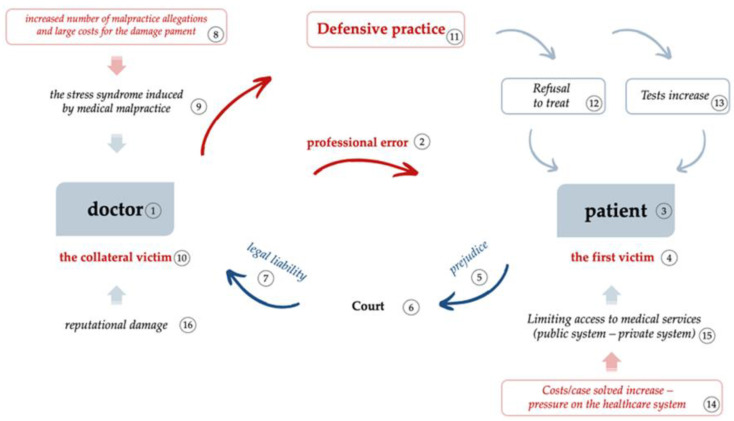
Schematic representation of the doctor–patient relationship, the factors that trigger DMP, and its consequences.

The phenomenon of defensive medical practice can be schematically illustrated by the factors that trigger it and the consequences it has on doctors, patients, and healthcare systems (Figure 10).

As shown in Figure 10, in the doctor *(1)*–patient *(3)* relationship, the constitutive elements of a professional error *(2)* intervene, regardless of the type of error—medical or ethical. The professional error generates damage to the treated patient, which places the patient in the category of *“first victim” (4)*. This patient, upon identifying the damage *(5)* caused by the performed medical act, has the legal capacity to address the court *(6)* and to trigger the legal liability of the doctor *(7)* via the right of access to justice, in order to obtain compensation for the damages suffered.

As a result of the malpractice claim made by the injured patient, the increase in accusations against doctors, and the large amounts of payments made to injured patients *(8)*, a “clinical–judicial syndrome” is developing at the medical community level, which in turn generates *“the stress syndrome induced by medical malpractice” (9)*. Doctors’ desires to protect themselves from possible litigation and its adverse effects (behavioral changes, isolation, depression and anxiety, effort, lost time, damaged reputation), push the doctors into defensive medical practices. In Figure 10, this phenomenon places the doctor in the position of a “collateral (secondary) victim” *(10)* of this complex process [10]. It is important to be aware that the defensive practice phenomenon *(11)* leads to a doctor’s sense of victimization and the doctor will, in turn, react by refusing to treat *(12)* or by unjustifiably increasing the number of medical investigations *(13)*, actions that increase costs/litigation *(14)* and make patients vulnerable by limiting their access to medical services *(15)*. Theses limitation impact both the public and private systems of financing healthcare services. In the first case, the limitations result in treatment of a smaller number of patients relative to the same financial resources. In the second situation, the limitation refers the access of patients who do not have sufficient financial resources to cover the costs of treatment, which increase due to the increased number of investigations. At the same time, the increase in costs/ litigation results in a requirement for larger numbers of medical personnel and the rapid consumption of allocated financial resources, with restricted access to treatment for people who have justified needs for medical services.

Defensive medicine is a phenomenon that affects the functionality of a healthcare system. Initially, the blame for triggering this phenomenon rests exclusively on the doctor/specialist who acts defensively. However, identifying a main culprit and continuously blaming that person do not diminish the defensive medical phenomenon or its consequences.

Analyzing the way in which the phenomenon of defensive practice works, its characteristic elements, and the multitude of factors that trigger and maintain this phenomenon, it is clear that a remedy may be found in changes to health systems by adapting and improving the doctor–patient relationship together with changes in the legal system by creating efficient mechanisms for settling claims resulting from alleged acts of malpractice, so as to ensure the appropriate compensation for the first victim—the patient—without turning the doctor involved into a collateral victim.

## Figures and Tables

**Table 1:** Comparative analysis of the fault system and the no-fault system.

Fault System	No-Fault System
System applied in most countries of the world (the United States of America, member states of the European Union, Romania).	System implemented in the Scandinavian countries, in Sweden since 1975, in France since 2002 (mixed system).
System focused on the search for the mistake and proof.	It is not necessary to prove the error in order for the injured party to receive compensation.
System focused on the causal links among act—damage—guilt.	System focused on the direct satisfaction of the injured patient.
Conflict character prevails, specific to a judicial procedure.	Settlement through extrajudicial administrative procedures.
High procedural costs.	Low procedural costs.
Long settlement terms.	Fast resolution.
Publicity for malpractice cases.	Increased confidentiality for malpractice cases.

## Data Availability

Not applicable.
